# Journey From Acute In-Patient to Community-Based Mental Health Rehabilitation: Outcome of Ayu-Psychiatry Care Initiative

**DOI:** 10.1192/bjo.2022.207

**Published:** 2022-06-20

**Authors:** Neha Sharma, Maël Voegeli, Venkata Joshi, Skanthesh Lakshmanan

**Affiliations:** 1 Aarogyam (UK) CIC, Leicester, United Kingdom; 2 VP Research Foundation, Coimbatore, India; 3 Croydon Ayurveda Centre, London, United Kingdom; 4 NMP Medical Research Institute, Jaipur, India

## Abstract

**Aims:**

In developing countries specially in sub-urban or rural areas, most patients with psychiatric crisis phase don't access intensive care. In India, AYUSH system of medical care is widely used, including crisis resolution and community treatment. However, evidence to support their effectiveness has remained very low. Present study is designed as community based participatory research, where Ayurveda management from acute in-patient care to a community-focused treatment and rehabilitation was studied.

**Methods:**

In this evaluation study, we trace the journey of Ayu-Psychiatry Care project, set up as community based mental health rehabilitation program in rural and sub-urban areas of Rajasthan, India, from acute in-patient care to a community-focused treatment and rehabilitation.

**Results:**

While receiving Ayu-Care and promoting early treatment and rehabilitation, community-based treatment demonstrated considerable improvement in maintaining family relationships and employment. Increased treatment adherence, improved self-efficacy, and reduced stigma were all made possible because to this community-based strategy.

**Conclusion:**

The connection between UK and Indian organisations is also explored during the journey. The findings of the study and the principles of long-term international cooperation are laid out by the authors.

